# Supplementary material for: Oral PRI-002 treatment in patients with MCI or mild AD: a randomized, double-blind phase 1b trial
Source: Nat Commun. 2025 May 6;16:4180. doi: 10.1038/s41467-025-59295-z (PMC12053642; doi:10.1038/s41467-025-59295-z)
Supplement: Supplementary file 1 — Supplementary Information [file 41467_2025_59295_MOESM1_ESM.pdf]

SUPPLEMENTARY INFORMATION

Oral PRI-002 treatment in patients with MCI or mild AD: a randomized, double-blinded phase 1b trial.

Authors: Janine Kutzsche[1], Nicoleta Carmen Cosma[2,3], Gunther Kauselmann[1], Friederike Fenski[2], Christine Bieniek[2], Tuyen Bujnicki[1], Marlene Pils[4], Oliver Bannach[4], Dieter Willbold[1,5*], Oliver Peters[2*]

	**Screening**	**Ambulant Phase**					**Follow-up EoS**
**Day of treatment**	**-21 to -1**	**1 Admission/ Baseline**	**7 (±1) Phone visit**	**14 (±1)**	**21 (±1) Phone visit**	**28 (±1) EoT**	**56 (±1)**
Informed consent	X						
Inclusion/Exclusion criteria	X	X					
Demography and medical history	X						
Physical exam	X	X		X		X	X
[1] Vital signs, weight	X	X		X		X	
[2] ECG (12-lead)		X		X		X	
EEG	X					X	
Concomitant medications	X	X	X	X	X	X	X
Adverse events assessment		X	X	X	X	X	X
CSF		X				X	
Feces (optional)		X				X	
Dispense trial medication		X		X		X	
[3] MRI	X					X	X
**Cognitive Testing**							
MMSE	X						
CERAD+		X				X	X
CDR		X				X	X
**Laboratory Tests**							
Safety laboratory	X	X		X		X	X
Urinalysis	X						
[4] Pharmacokinetic sampling		X				X	

[1] Heart rate and blood pressure: Day 1: at pre-dose and 2 hours±15 minutes; Day 14 and 28: at pre-dose; [2] ECG Day 1: at pre-dose and 2 hours ± 15 minutes; Day 14 and 28: at pre-dose; [3] Safety MRI was scheduled at screening and within three days after Day 28 and Day 56;[4] Pharmacokinetic sampling Day 1 and Day 28: at pre-dose, 30 minutes ± 15 minutes, 1 hour ± 15 minutes, 2 hours ± 15 minutes, and 4 hours ± 15 minutes.

CDR: Clinical dementia rating; CERAD: Consortium to Establish a Registry for Alzheimer's Disease; CSF: Cerebrospinal fluid; ECG: Electrocardiogram; EEG: Electroencephalogram; EoS: End-of-Study; EoT: End-of-Treatment; MMSE: Mini-Mental State Examination; MRI: Magnetic resonance imaging.

SupplementaryTable 1: **Study Schedule of Activities**

Clinical event according to MedDRA version 23.0	GRADE			TOTAL
	1 (Mild)	2 (Moderate)	3 (Severe)	
**PRI-002 (n=9)**				
Number of subjects with at least one AE	4	1	0	5
Total number of AEs	14	2	0	16
Gastrointestinal disorders	1	0	0	1
Musculoskeletal and connective tissue disorders	1	0	0	1
Nervous system disorders	5	2	0	7
Injury, poisoning and procedural complications	1	0	0	1
General disorders and administration site disorders	1	0	0	1
Vascular disorders	1	0	0	1
Psychiatric disorders	3	0	0	3
Infections and infestations	1	0	0	1
**Placebo (n = 10)**				
Number of subjects with at least one AE	7	1	0	8
Total number of AEs	26	1	0	27
Psychiatric disorders	5	0	0	5
Nervous system disorders	1	0	0	1
Gastrointestinal disorders	3	0	0	3
Skin and subcutaneous tissue disorders	2	0	0	2
Musculoskeletal and connective tissue disorders	4	0	0	4
Vascular disorders	5	0	0	5
Injury, poisoning and procedural complications	2	1	0	3
General disorders and administration site conditions	1	0	0	1
Ear and labyrinth disorders	1	0	0	1
Metabolism and nutrition disorders	1	0	0	1
Infections and infestations	1	0	0	1

Supplementary Table 2: Overall Incidence of Adverse Events according to system organ class (MedDRA)

Parameters	Treatment	Screening (Day 0)	Baseline (Day 1)	(Day 14)	EoT (Day 28)	FU EoS (Day 56)
**Hematology**						
Leucocyte [1/nl]	Placebo	5.82±2.65	5.47±1.74	5.38±2.17	5.52±2.09	5.64±1.69
	PRI-002	6.31±2.13	6.00±1.89	5.95±1.71	5.86±1.81	5.66±1.41
Platelets [1/nl]	Placebo	252.97±77.74	240.13±70.03	246.55±71.39	229.59±73.35	239.97±73.53
	PRI-002	230.44±43.87	206.25±39.51	232.22±44.84	227.02±49.58	240.06±48.46
Hemoglobin [g/dl]	Placebo	13.68±0.84	13.24±0.77	13.56±0.82	13.04±0.82	13.73±0.97
	PRI-002	13.67±1.15	13.34±0.99	13.44±1.09	13.21±0.98	13.27±1.03
Hematocrit [l/l]	Placebo	0.40±0.02	0.39±0.03	0.40±0.03	0.38±0.02	0.41±0.03
	PRI-002	0.39±0.03	0.38±0.03	0.39±0.03	0.38±0.03	0.40±0.03
Erythrocyte [1/pl]	Placebo	4.43±0.28	4.33±0.25	4.40±0.25	4.27±0.24	4.47±0.26
	PRI-002	4.21±0.39	4.13±0.38	4.14±0.42	4.06±0.44	4.10±0.50
**Clinical chemistry**						
Sodium [mmol/l]	Placebo	140.18±2.44	140.31±2.74	141.98±2.71	140.49±1.90	141.89±2.13
	PRI-002	140.65±2.45	140.36±2.50	139.65±2.45	139.77±1.99	139.87±2.20
Potassium [mmol/l]	Placebo	4.25±0.30	4.52±0.64	4.28±0.42	4.05±0.26	4.07±0.45
	PRI-002	4.39±0.41	4.22±0.39	4.11±0.20	4.21±0.46	4.06±0.27
ALT [U/l]	Placebo	18.12±6.51	16.92±6.14	17.30±9.40	16.90±5.04	15.85±7.13
	PRI-002	28.02±15.21	24.70±13.73	27.05±18.50	27.19±15.89	23.94±22.30
AST [U/l]	Placebo	24.62±5.30	28.77±12.27	23.37±6.35	24.26±5.27	23.36±5.83
	PRI-002	30.33±7.91	31.22±12.08	29.23±10.56	30.35±7.57	29.86±24.32
CRP [mg/l]	Placebo	0.80±0.30	1.19±0.48	0.69±0.10	0.99±0.87	1.11±0.38
	PRI-002	2.40±3.71	1.92±2.51	1.93±3.19	1.67±3.87	1.82±3.69
Creatine kinase [U/l]	Placebo	81.42±41.99	90.32±46.34	78.87±27.82	80.48±32.98	74.42±30.42
	PRI-002	104.03±95.19	104.42±138.05	105.33±85.41	113.95±94.37	101.19±42.07
Creatinine [mg/dl]	Placebo	0.87±0.29	0.88±0.18	0.89±0.17	0.85±0.19	0.90±0.20
	PRI-002	0.86±0.28	0.88±0.31	0.85±0.25	0.86±0.29	0.86±0.29
**Hematoserology**						
INR [%]	Placebo	102.22±14.32	102.16±14.93	102.14±15.24	97.58±13.27	103.56±12.16
	PRI-002	97.31±21.57	104.48±9.63	96.99±21.30	101.13±9.55	98.71±21.08
PTT [s]	Placebo	30.58±4.60	30.48±4.21	31.97±3.90	32.74±2.63	32.76±4.81
	PRI-002	30.24±4.42	29.05±2.22	31.60±3.95	29.71±1.94	30.96±3.11

Values are provided as geometric mean ± SD.
EoT: End of treatment; EoS: end of study; ALT: alanine aminotransaminase; AST: aspartate aminotransferase; CRP: creatinine reactive protein; INR: International Normalized Ratio.

Supplementary Table 3: Laboratory Parameter

Parameter	Statistics	300 mg PRI-002	
		Day 1 (n=9)	Day 28 (n=8)
C_{max} (ng/mL)	Mean ± SD	4.46 ± 3.71	12.6 ± 13.2
	CV (%)	83	105
	Median	3.03	6.2
	Min	0.7	1.8
	Max	10.4	32.9
T_{max} (h)	Mean ± SD	1.94 ± 1.61	1.1 ± 1.3
	CV (%)	83	113
	Median	1.0	0.5
	Min	0.5	0.5
	Max	4.0	4.0
$AUC_{0\text{-}4\ hours}$ (ng*h/ml)	Mean ± SD	9.98 ± 7.46	26.8 ± 23.8
	CV (%)	75	89
	Median	8.5	20.4
	Min	2.0	5.9
	Max	22.0	70.9
$AUC_{0\text{-}4}$ (Day28/Day1)	Mean ± SD		2.5 ± 1.3
	CV (%)		53
	Median		2.8
	Min		0.6
	Max		4.0
Css trough	Mean ± SD		1.97 ± 2.44
	CV (%)		134
	Median		1.27
	Min		0.0
	Max		7.45

$AUC_{0\text{-}4}$: Area under the plasma concentration-time curve from time zero to four hours: C_{max}: Maximum plasma concentration; Css: Concentration of drug in plasma at steady state; Css trough: trough concentration at steady state CV: Coefficient of variation; Max: Maximum; Min: Minimum; n: Number; SD: Standard deviation; T_{max}: Time to reach maximum plasma concentration.

Supplementary Table 4: Pharmacokinetic parameters

Variable	*Day*	*Placebo*			*PRI-002*			*PV*	*PV-LME*
		mean	*SD*	*N*	*mean*	*SD*	*N*		
TMT-A	1	45.3	13.5	10	60.4	26.7	9	0.24	0.49
	28	45.8	11.4	9	58.4	23.9	8	0.31	
	56	43.7	9.9	10	53.9	21.1	9	0.46	
TMT-B	1	121.5	43.7	10	157.6	72.1	9	0.27	0.23
	28	113.3	25.3	9	137.5	70.2	8	0.74	
	56	112.3	34.0	10	121.9	56.1	9	0.87	
Fig Draw	1	10.6	0.7	10	10.3	1.1	9	0.77	0.09
	28	10.9	0.3	9	10.8	0.7	8	0.93	
	56	10.6	1.0	10	10.9	0.3	9	0.61	
Fig Recall	1	4.2	3.2	10	5.7	3.4	9	0.37	0.64
	28	5.6	3.0	9	6.9	3.8	8	0.41	
	56	6.6	3.0	10	7.4	4.4	9	0.43	
Clock	1	1.6	0.8	10	1.7	0.9	9	0.89	0.78
	28	1.6	0.9	9	1.6	1.1	8	0.95	
	56	1.7	0.8	10	1.7	1.1	9	0.71	
WL learn	1	14.3	3.7	10	15.4	4.6	9	0.74	0.04
	28	15.3	4.3	9	17.3	5.0	8	0.92	
	56	15.5	3.4	10	19.3	4.7	9	0.04	
WL Recall	1	2.4	1.7	10	4.7	2.3	9	0.04	0.17
	28	3.2	2.2	9	6.0	3.3	8	0.07	
	56	4.2	1.4	10	5.4	2.2	9	0.39	
WMS delayed recall	1	2.1	2.3	10	10.0	11.6	9	0.09	0.58
	28	3.3	6.0	9	12.8	12.0	8	0.05	
	56	5.8	6.1	10	15.0	13.6	9	0.14	

P-values for one time point (column 'PV') were calculated using two-sided Wilcoxon test. P-values for the comparison of longitudinal effects between both groups (Placebo vs. PRI-002) (column 'PVLME') where calculated with mixed linear models. Day1 is the baseline visit, day 28 is the end of treatment visit and day 56 is the end of study visit. TMT-A: Tests Trail Making Test A, TMT-B: Tests Trail Making Test B, Fig Draw: Figure Drawing test, Fig Recall: Figure Recall test, Clock: Clock drawing test, WL learn: word list learning test, WL Recall: Word list recall test, WMS: Wechsler Memory Scale delayed recall test.

Supplementary Table 5 with statistics for CERAD variables.

Patient	Treatment	Difference WL learn<br><br>Baseline (day 1) to EoS (day 56)	Duration cholinesterase inhibitor treatment [month]	Diagnosis
1	PRI-002	7	3	MCI
2	PRI-002	4		MCI
3	PRI-002	2	10	MCI
4	PRI-002	5		MCI
5	PRI-002	1		MCI
6	PRI-002	4	3	MCI
7	PRI-002	6	30	AD
8	PRI-002	3	3	MCI
9	PRI-002	3	4	AD
			Σ 53	
1	Placebo	-3	7	MCI
2	Placebo	0	3	MCI
3	Placebo	5		MCI
4	Placebo	0	6	MCI
5	Placebo	6		MCI
6	Placebo	-2	3	MCI
7	Placebo	2	3	MCI
8	Placebo	5	3	MCI
9	Placebo	-2	3	MCI
10	Placebo	1	3	MCI
			Σ 31	

Supplementary Table 6 Cholinesterase inhibitor treatment: Difference WL learn, Duration cholinesterase inhibitor treatment and Diagnosis, Σ: Total months of treatment

Patient PRI-002	ng PRI-002/ml
**1**	<0.200
**2**	0.203
**3**	0.377
**4**	0.304
**5**	<0.200
**6**	<0.200
**7**	<0.200
**8**	<0.200
**9**	<0.200

LLOQ:0.200; <0.200: below LLOQ

Supplementary Table 7 PRI-002 Level in CSF of individual patients at Day 28

	Mean (n=9)	SD	CV
**Hours**	**ng/ml**	**ng/ml**	**%**
	**Day 1**		
**0**	0	0	
**0.5**	3.1	4.2	136
**1**	3.5	2.9	84
**2**	2.6	1.6	62
**4**	1.9	1.2	65
	**Mean (n=8)**	**SD**	**CV**
**Hours**	**ng/ml**	**ng/ml**	**%**
	**Day 28**		
**0**	2.0	2.4	124
**0.5**	12.5	13.3	107
**1**	8.4	8.3	99
**2**	6.1	5.3	86
**4**	4.6	3.8	84

Supplementary Table 8 Descriptive Statistics (Mean, SD: standard deviation and CV: Coefficient of variation) of PRI-002 plasma levels at Day 1 and Day 28

Placebo Patient number+ A2:N42		Glucose	Glucose Outside expected range	Leucozytes	Leucozytes outside expected range	Nitrite	Nitrite outside expected range	Protein	Protein outside expected range	Erythrocytes	Erythrocytes outside expected range	HB	HB outside expected range
1	Baseline	Negativ		Negativ		Negativ		Negativ		Negativ		Negativ	
	Day 14	Negativ		Negativ		Negativ		Negativ		Negativ		Negativ	
	Day 28	Negativ		Negativ		Negativ		Negativ		Negativ		Negativ	
	Day 56	Negativ		Negativ		Negativ		Negativ		Negativ		Negativ	
2	Baseline	Negativ		Negativ		Negativ		Negativ		Negativ		Negativ	
	Day 14	Negativ		1+		Negativ		Negativ		Negativ		Negativ	
	Day 28	Negativ		Negativ		Negativ		1+		1+		Negativ	
	Day 56	Negativ		Negativ		Negativ		Negativ		Negativ		Negativ	
3	Baseline	Negativ		Negativ		Negativ		Negativ		Negativ		Negativ	
	Day 14	Negativ		Negativ		Negativ		Negativ		Negativ		Negativ	
	Day 28	Negativ		Negativ		Negativ		Negativ		Negativ		Negativ	
	Day 56	Negativ		Negativ		Negativ		Negativ		Negativ		Negativ	
4	Baseline	Negativ		Negativ		Negativ		Negativ		Negativ		Negativ	
	Day 14	Negativ		Negativ		Negativ		Negativ		Negativ		Negativ	
	Day 28	Negativ		Negativ		Negativ		Negativ		Negativ		Negativ	
	Day 56	Negativ		Negativ		Negativ		Negativ		Negativ		Negativ	
5	Baseline	Negativ		Negativ		Negativ		Negativ		Negativ		Negativ	
	Day 14	Negativ		Negativ		Negativ		1+	Yes	Negativ		Negativ	
	Day 28	Negativ		Negativ		1+		Negativ		Negativ		Negativ	
	Day 56	Negativ		Negativ		Negativ		Negativ		Negativ		Negativ	
6	Baseline	Negativ		Negativ		Negativ		Negativ		Negativ		Negativ	
	Day 14	Negativ		1+		Negativ		Negativ		Negativ		Negativ	
	Day 28	Negativ		Negativ		Negativ		Negativ		Negativ		Negativ	
	Day 56	Negativ		Negativ		Negativ		Negativ		Negativ		Negativ	
7	Baseline	Negativ		Negativ		Negativ		Negativ		Negativ		Negativ	
	Day 14	Negativ		Negativ		Negativ		Negativ		Negativ		Negativ	
	Day 28	Negativ		Negativ		Negativ		Negativ		1+		Negativ	
	Day 56	Negativ		Negativ		Negativ		Negativ		Negativ		Negativ	
8	Baseline	Negativ		Negativ		Negativ		Negativ		Negativ		Negativ	
	Day 14	Negativ		1+		Negativ		Negativ		Negativ		Negativ	
	Day 28	Negativ		Negativ		Negativ		Negativ		Negativ		Negativ	
	Day 56	Negativ		Negativ		Negativ		Negativ		Negativ		Negativ	
9	Baseline	Negativ		Negativ		Negativ		Negativ		Negativ		Negativ	
	Day 14	Negativ		Negativ		Negativ		Negativ		Negativ		Negativ	
	Day 28	Negativ		Negativ		Negativ		Negativ		Negativ		Negativ	
	Day 56	Negativ		1+		Negativ		Negativ		Negativ		Negativ	
10	Baseline	Negativ		Negativ		Negativ		Negativ		Negativ		Negativ	
	Day 14	Negativ		Negativ		Negativ		Negativ		Negativ		Negativ	
	Day 28	Negativ		1+		Negativ		Negativ		Negativ		Negativ	
	Day 56	Negativ		1+		Negativ		Negativ		Negativ		Negativ	

PRI-002 Patient number		Glucose	Glucose Outside expected range	Leucozytes	Leucozytes outside expected range	Nitrite	Nitrite outside expected range	Protein	Protein outside expected range	Erythrocytes	Erythrocytes outside expected range	HB	HB outside expected range
1	Baseline	Negativ		Negativ		Negativ		Negativ		Negativ		Negativ	
	Day 14	Negativ		Negativ		Negativ		Negativ		Negativ		Negativ	
	Day 28	Negativ		Negativ		Negativ		Negativ		Negativ		Negativ	
	Day 56	Negativ		Negativ		Negativ		Negativ		Negativ		Negativ	
2	Baseline	Negativ		Negativ		Negativ		1+	yes	Negativ		Negativ	
	Day 14	Negativ		Negativ		Negativ		Negativ		Negativ		Negativ	
	Day 28	Negativ		Negativ		Negativ		1+		Negativ		Negativ	
	Day 56	Negativ		Negativ		Negativ		Negativ		Negativ		Negativ	
3	Baseline	Negativ		Negativ		Negativ		Negativ		Negativ		Negativ	
	Day 14	Negativ		Negativ		Negativ		Negativ		Negativ		Negativ	
	Day 28	Negativ		Negativ		Negativ		Negativ		Negativ		Negativ	
	Day 56	Negativ		Negativ		Negativ		Negativ		Negativ		Negativ	
4	Baseline	Negativ		Negativ		Negativ		Negativ		1+		Negativ	
	Day 14	Negativ		Negativ		Negativ		Negativ		Negativ		Negativ	
	Day 28	Negativ		Negativ		Negativ		Negativ		Negativ		Negativ	
	Day 56	Negativ		Negativ		Negativ		Negativ		2+	yes	Negativ	
5	Baseline	Negativ		Negativ		Negativ		Negativ		Negativ		Negativ	
	Day 14	Negativ		Negativ		Negativ		Negativ		Negativ		Negativ	
	Day 28	Negativ		Negativ		Negativ		Negativ		Negativ		Negativ	
	Day 56	Negativ		Negativ		Negativ		Negativ		Negativ		Negativ	
6	Baseline	Negativ		Negativ		Negativ		Negativ		Negativ		1+	
	Day 14	Negativ		Negativ		1+	yes	Negativ		1+	yes	Negativ	
	Day 28	Negativ		Negativ		Negativ		Negativ		2+		Negativ	
	Day 56	Negativ		Negativ		Negativ		Negativ		Negativ		Negativ	
7	Baseline	Negativ		Negativ		Negativ		1+		Negativ		Negativ	
	Day 14	Negativ		Negativ		Negativ		Negativ		Negativ		Negativ	
	Day 28	Negativ		Negativ		Negativ		Negativ		Negativ		Negativ	
	Day 56	Negativ		Negativ		Negativ		Negativ		Negativ		Negativ	
8	Baseline	Negativ		Negativ		Negativ		Negativ		Negativ		Negativ	
	Day 14	Negativ		Negativ		Negativ		Negativ		Negativ		Negativ	
	Day 28	Negativ		Negativ		Negativ		Negativ		Negativ		Negativ	
	Day 56	Negativ		Negativ		Negativ		Negativ		Negativ		Negativ	
9	Baseline	2+	yes	1+	yes	Negativ		1+	yes	Negativ		Negativ	
	Day 14	Negativ		Negativ		Negativ		Negativ		Negativ		Negativ	
	Day 28	2+		2+		Negativ		1+		Negativ		Negativ	
	Day 56	Negativ		Negativ		Negativ		Negativ		Negativ		Negativ	

Supplementary Table 9 Urinalysis

HB: hemoglobin

PR+B3:J43I-002 Patient number		Height [cm]:	Weight [kg]:	BMI [kg/m²]:	Body temperatur e [C°]:	Blood pressure systolic [mm Hg]:	Blood pressure diastolic [mmHg]:	Heart beat [1/min]:
1	Screening	165	56,5	20,8	36,2	121	76	54
	Baseline		56,3	20,7	36,5	112	76	64
	Day 28		55,6	20,4	36,2	139	87	56
	Day 14		57,2		36,2	125	71	59
	Day 56		55,2		36	138	86	59
2	Screening	180	73,5	22,7	36,5	120	71	58
	Baseline		73,4	22,7	36,7	114	66	65
	Day 14		73,2	22,6	36,4	107	68	69
	Day 28		72,9	22,5	36,6	101	66	67
	Day 56		72,5	22,4	36,8	105	58	68
3	Screening	153	49	20,9	37	143	73	77
	Baseline		49,8	21,3	36,3	134	76	78
	Day 14		50,2	21,4	36,8	125	70	62
	Day 28		49,4	21,1	36,7	138	75	77
	Day 56		48,5	20,7	36,3	126	80	78
4	Screening	171	67,5	23,1	37	124	91	77
	Baseline		66,6	22,8	37,1	129	81	68
	Day 14		67,5	23,1	36,9	154	96	73
	Day 28		67,6	23,1	36,9	153	84	63
	Day 56		66,7	22,8	36,9	136	78	64
5	Screening	182	83,4	25,2	36,4	144	72	46
	Baseline		82,6	24,9	36,3	152	72	60
	Day 14		83,5	25,2	36,8	142	82	54
	Day 28		83,5	25,2	36,9	139	71	58
	Day 56		82,7	25	37,1	126	67	57
6	Screening	153	44,9	19,2	36,4	136	71	74
	Baseline		44,8	19,1	37,4	147	73	84
	Day 14		44,4	19	36,7	127	79	77
	Day 28		44,8	19,1	36,7	138	77	81
	Day 56		46,8	20	36,9	139	78	84
7	Screening	176	78,7	25,4	36,6	131	74	59
	Baseline		78,5	25,3	37	103	73	71
	Day 14		80,9	26,1	36,6	108	72	70
	Day 28		80,5	26	36,9	117	70	65
	Day 56		79	25,5	36,8	111	65	63
8	Screening	182	83,2	25,1	36,9	128	65	62
	Baseline		83,9	25,3	36,6	117	71	81
	Day 14		83	25,1	36,5	112	68	62
	Day 28		83,7	25,3	36,4	112	63	78
	Day 56		82,2	24,8	36,7	110	63	73
9	Screening	157	78,5	31,8	36,7	112	82	97
	Baseline		78,9	32	36,4	122	84	104
	Day 14		82,9	33,6	36,2	117	97	108
	Day 28		79,1	32,1	36,2	135	94	99
	Day 56		80,2	32,5	35,8	124	83	100

Placebo Patient number		Height [cm]:	Weight [kg]:	BMI [kg/m²]:	Body temperature [C°]:	Blood pressure systolic [mm Hg]:	Blood pressure diastolic [mmHg]:	Heart beat [1/min]:
1	Baseline		70	22,1	35,9	165	83	49
	Day 14		72,7	22,9	35,2	137	71	62
	Screening	178	69,8	22	35,8	147	76	39
	Day 28		72,6	22,9	36,3	142	86	53
	Day 56		72,6	22,9	35,7	146	85	71
2	Screening	161	60,7	23,4	36,3	129	83	63
	Baseline		60	23,1	36,5	153	93	70
	Day 14		62,7	24,2	36,5	129	77	61
	Day 28		60,5	23,3	36,7	126	80	69
	Day 56		61,7	23,8	36,6	136	79	59
3	Screening	167	62,9	22,6	36,8	133	81	73
	Baseline		62,5	22,4	36,3	120	81	81
	Day 14		63,9	22,9	36,8	121	75	72
	Day 28		62,8	22,5	37	126	77	63
	Day 56		62,6	22,4	36,4	140	83	70
4	Screening	161	55	21,2	35,6	131	76	66
	Baseline		55,2	21,3	36,5	141	77	75
	Day 14		53,7	20,7	36,6	143	81	69
	Day 28		54,2	20,9	36,1	121	72	68
	Day 56		55,5	21,4	36	141	86	59
5	Screening	159	55	21,8	36,5	116	77	70
	Baseline		53,2	21	35,8	111	76	81
	Day 14		53,4	21,1	36,2	148	79	68
	Day 28		53,3	21,1	36,8	130	91	75
	Day 56		53,6	21,2	36,9	111	72	72
6	Screening	161	62,3	24	36,2	140	86	78
	Baseline		62,3	24	36,9	142	99	74
	Day 14		62,8	24,2	36,7	163	97	93
	Day 28		62,7	24,2	36	154	100	87
	Day 56		64,4	24,8	36,5	131	82	84
7	Screening	174	64	21,1	36,4	140	85	72
	Baseline		64	21,1	36,4	150	90	74
	Day 14		63,1	20,8	36,9	134	74	66
	Day 28		61	20,1	36,5	141	77	67
	Day 56		63,6	21	36,1	151	88	67
8	Screening	150	61,8	27,5	36,9	149	79	74
	Baseline		61,7	27,4	36,7	170	90	75
	Day 14		60,6	26,9	37,1	162	87	88
	Day 28		59,7	26,5	36,7	162	86	72
	Day 56		66,7	29,6	36,8	156	87	85
9	Day 14		65	21,7	36,8	104	77	66
	Screening	173	63	21	36,1	123	64	60
	Baseline		63,1	21,1	36,2	135	62	71
	Day 28		63,3	21,2	36,3	138	68	77
	Day 56		62,8	21	36,6	126	62	74
10	Screening	158	63,5	25,4	36,6	136	74	63
	Baseline		64	25,6	37,2	142	75	63
	Day 14		62	24,8	36,9	127	71	64
	Day 28		64,2	25,7	37,1	119	66	70
	Day 56		64,8	26	37	149	76	68

Supplementary Table 10 Vital signs. BMI: body mass index

PRI-002 Patient number		[Heart beat] Value:	[PQ-Interval] Value:	[PQ-Interval] Value not	[QRS] Value:	[QTc] Wert:	Result:	General comments:
1	Baseline	60		Yes	92	428	Normal	
	Day 28	60		Yes	97	432	Not normal, clinically not relevant	
	Day 14	60		Yes	102	429	Not normal, clinically not relevant	
2	Baseline	63		Yes	94	431	Not normal, clinically not relevant	
	Day 14	60		Yes	98	417	Not normal, clinically not relevant	
	Day 28	61		Yes	94	408	Normal	
3	Baseline	70	182		74	430	Not normal, clinically not relevant	
	Day 14	58	198		76	406	Normal	
	Day 28	72		Yes	82	423	Normal	
4	Baseline	63	140		88	403	Normal	
	Day 14	57		Yes	85	390	Normal	
	Day 28	59	132		90	408	Normal	
5	Baseline	57	124		155	468	Not normal, clinically not relevant	Right bundle branch block; left anterior hemiblock; Sinusbradycardia
	Day 14	52	145		157	448	Not normal, clinically not relevant	
	Day 28	48		Yes	155	438	Not normal, clinically not relevant	
6	Baseline	84		Yes	87	406	Normal	
	Day 14	74		Yes	89	405	Normal	
	Day 28	73	122		90	406	Normal	
7	Baseline	65		Yes	88	372	Normal	
	Day 14	63		Yes	97	373	Not normal, clinically not relevant	
	Day 28	61	157		87	374	Normal	
8	Baseline	78	150		110	403	Normal	
	Day 14	59	162		103	376	Not normal, clinically not relevant	Sinusbradycardia, HF 59/Min.
	Day 28	68	156		102	401	Not normal, clinically not relevant	
9	Baseline	102	182		98	419	Not normal, clinically not relevant	Sinustachycardia; HF 102/Min. no ERBST
	Day 14	100	182		95	424	Not normal, clinically not relevant	
	Day 28	94	183		84	424	Normal	

Placebo Patient number		[Heart beat] Value:	[PQ-Interval] Value:	[PQ-Interval] Value not available:	[QRS] Value:	[QTc] Wert:	Result:	General comments:
1	Baseline	46		Yes	105	408	Not normal, clinically not relevant	
	Day 14	55		Yes	116	420	Not normal, clinically not relevant	
	Day 28	49		Yes	104	410	Not normal, clinically not relevant	
2	Baseline	55	208		80	392	Not normal, clinically not relevant	
	Day 14	65		Yes	68	377	Not normal, clinically not relevant	
	Day 28	65		Yes	101	387	Not normal, clinically not relevant	
3	Baseline	69	126		100	452	Not normal, clinically not relevant	
	Day 14	63	132		109	432	Not normal, clinically not relevant	
	Day 28	60		Yes	105	420	Not normal, clinically not relevant	
4	Baseline	64	190		90	388	Normal	
	Day 14	69	197		85	372	Normal	PQ borderline (<200)
	Day 28	60		Yes	84	374	Normal	
5	Baseline	64	152		76	375	Normal	
	Day 14	55		Yes	84	378	Not normal, clinically not relevant	
	Day 28	72	157		85	385	Normal	
6	Baseline	74	217		80	401	Not normal, clinically not relevant	
	Day 14	81		Yes	84	410	Normal	
	Day 28	80		Yes	84	422	Not normal, clinically not relevant	ventricular extra systoles
7	Baseline	67	156		90	416	Normal	
	Day 14	65		Yes	94	412	Normal	
	Day 28	67		Yes	90	409	Normal	
8	Baseline	72	141		81	416	Normal	
	Day 14	72		Yes	79	393	Normal	
	Day 28	64	146		77	374	Normal	
9	Day 14	60	171		106	404	Normal	
	Baseline	65	165		110	413	Not normal, clinically not relevant	
	Day 28	62	173		102	412	Normal	
10	Baseline	64		Yes	86	396	Normal	
	Day 14	60		Yes	85	396	Not normal, clinically not relevant	
	Day 28	66	221		77	376	Not normal, clinically not relevant	SR, HF 667min, incompl. RSB, otherwise no finding

Supplementary Table 11 ECG parameters.

PRI-002 Patient number+B3: E38		Result:	Clinically significant
1	Screening	Normal	
	Day 28	Normal	
2	Screening	Normal	
	Day 28	Normal	
3	Screening	Normal	
	Day 28	Normal	
4	Day 28	Normal	
	Screening	Normal	
5	Screening	Normal	
	Day 28	Normal	
6	Day 28	Normal	
	Screening	Normal	
7	Screening	Normal	
	Day 28	Normal	
8	Screening	Normal	
	Day 28	Normal	
9	Screening	Normal	
	Day 28	Normal	

Placebo Patient number		Result:	Clinically significant
1	Screening	Normal	
	Day 28	Normal	
2	Screening	Normal	
	Day 28	Normal	
3	Screening	Abnormal	No
	Day 28	Abnormal	No
4	Screening	Normal	
	Day 28	Normal	
5	Screening	Normal	
	Day 28	Normal	
6	Screening	Normal	
	Day 28	Normal	
7	Screening	Normal	
	Day 28	Normal	
8	Screening	Normal	
	Day 28	Normal	
9	Screening	Abnormal	No
	Day 28	Normal	
10	Screening	Normal	
	Day 28	Normal	

Supplementary Table 12 EEG results.

PRI-002 Patient number		Age appropriate	If no, please specify:	Clinically noticeable	In accordance with diagnosis	New aspects compared to preliminary consultation	If yes, please specify:	Relation to study medication	General comments:
1	Screening	Yes							
	Day 28					No		No	
	Day 56					No		No	
2	Screening	Yes							
	Day 28					No		No	
	Day 56					No		No	
3	Screening	Yes							Chronic hemorrhagic transformed thalamic infarction left. biparietal atrophy, MTA 1-2 bds.
	Day 28					No		No	
	Day 56					Yes	Micro hemorrhage in the left superior temporal gyrus	No	
4	Screening	Yes							known chronic sinusitis, known temporal atrophy (MTA2) in Alzheimer's disease
	Day 28					No		No	Beginning right medial temporal atrophy
	Day 56					No		No	
5	Screening	Yes							Microhemorrhage left temporal, left-sided medial temporal atrophy
	Day 28					No		No	
	Day 56					Yes	Scheltens score 1-2	No	
6	Screening	Yes							
	Day 28					No		No	
	Day 56					No		No	
7	Screening	Yes							
	Day 28					No		No	
	Day 56					No		No	
8	Screening	Yes							
	Day 28					No		No	
	Day 56					No		No	
9	Screening	Yes							
	Day 28					No		No	
	Day 56					No		No	

Placebo Patient number		Age appropriate	If no, please specify:	Clinically noticeable	In accordance with diagnosis	New aspects compared to preliminary consultation	If yes, please specify:	Relation to study medication	General comments:
1	Screening	No	Bifrontobasal defekt of unclear etiology	No					
	Day 28					No		No	
	Day 56					No		No	
2	Screening	No	Leuk encephalopathie	No					
	Day 28					No		No	
	Day 56					No		No	
3	Screening	No	MTA Score 3 (Hippocampus atrophy) 5mm	Yes	Yes				
	Day 28					No		No	Mikrohemorrhages biparietal emphasized brain
	Day 56					Yes	Newly occurring left	No	
4	Screening	Yes							Known hippocampal atrophy (MTA3)
	Day 28					No		No	
	Day 56					Yes	Micro hemorrhage right thalamus	No	
5	Screening	Yes							
	V6 FU EoS (D56)					No		No	
	Day 28					No		No	
6	Screening	Yes							
	Day 28					Yes	Micro hemorrhage right parietal	No	
	Day 56					No		No	
7	Screening	Yes							Two pinpoint microhemorrhages in the pons on the right and left. Flair hyperintense cortical signal left occipital
	Day 28					No		No	
	Day 56					No		No	
8	Screening	Yes							Bi-parietal accentuated early brain involution
	Day 28					No		No	
	Day 56					No		No	
9	Screening	Yes							
	Day 28					No		No	
	Day 56					No		No	
10	Screening	Yes							
	Day 28					No		No	
	Day 56					No		No	

Supplementary Table 13 MRI results.
