# Supplementary material for: Oral PRI-002 treatment in patients with MCI or mild AD: a randomized, double-blind phase 1b trial
Source: Nat Commun. 2025 May 6;16:4180. doi: 10.1038/s41467-025-59295-z (PMC12053642; doi:10.1038/s41467-025-59295-z)
Supplement: Supplementary file 2 — Reporting Summary [file 41467_2025_59295_MOESM2_ESM.pdf]

nature portfolio

Corresponding author(s): NCOMMS-24-73793-T

Last updated by author(s): Oct 29, 2024

Reporting Summary

Nature Portfolio wishes to improve the reproducibility of the work that we publish. This form provides structure for consistency and transparency in reporting. For further information on Nature Portfolio policies, see our Editorial Policies and the Editorial Policy Checklist.

Statistics

For all statistical analyses, confirm that the following items are present in the figure legend, table legend, main text, or Methods section.

n/a	Confirmed	
☐	☒	The exact sample size (*n*) for each experimental group/condition, given as a discrete number and unit of measurement
☐	☒	A statement on whether measurements were taken from distinct samples or whether the same sample was measured repeatedly
☐	☒	The statistical test(s) used AND whether they are one- or two-sided<br>*Only common tests should be described solely by name; describe more complex techniques in the Methods section.*
☒	☐	A description of all covariates tested
☐	☒	A description of any assumptions or corrections, such as tests of normality and adjustment for multiple comparisons
☐	☒	A full description of the statistical parameters including central tendency (e.g. means) or other basic estimates (e.g. regression coefficient) AND variation (e.g. standard deviation) or associated estimates of uncertainty (e.g. confidence intervals)
☐	☒	For null hypothesis testing, the test statistic (e.g. *F*, *t*, *r*) with confidence intervals, effect sizes, degrees of freedom and *P* value noted<br>*Give P values as exact values whenever suitable.*
☒	☐	For Bayesian analysis, information on the choice of priors and Markov chain Monte Carlo settings
☒	☐	For hierarchical and complex designs, identification of the appropriate level for tests and full reporting of outcomes
☐	☒	Estimates of effect sizes (e.g. Cohen's *d*, Pearson's *r*), indicating how they were calculated

Our web collection on statistics for biologists contains articles on many of the points above.

Software and code

Policy information about availability of computer code

Data collection	*Provide a description of all commercial, open source and custom code used to collect the data in this study, specifying the version used OR state that no software was used.*
Data analysis	All statistical data analysis was performed with the certified software Statistical Analysis System (SAS) by MicroDiscovery GmbH (Berlin, Germany).

For manuscripts utilizing custom algorithms or software that are central to the research but not yet described in published literature, software must be made available to editors and reviewers. We strongly encourage code deposition in a community repository (e.g. GitHub). See the Nature Portfolio guidelines for submitting code & software for further information.

Data

Policy information about availability of data

All manuscripts must include a data availability statement. This statement should provide the following information, where applicable:

- Accession codes, unique identifiers, or web links for publicly available datasets
- A description of any restrictions on data availability
- For clinical datasets or third party data, please ensure that the statement adheres to our policy

Source data are provided with this paper.

Research involving human participants, their data, or biological material

Policy information about studies with human participants or human data. See also policy information about sex, gender (identity/presentation), and sexual orientation and race, ethnicity and racism.

Reporting on sex and gender	In the study 11 females and 8 males were included, so the findings apply to both sexes. Only sex was considered in study design and sex was determined based on self-reporting.
Reporting on race, ethnicity, or other socially relevant groupings	n/a
Population characteristics	In this study male and female (not of childbearing potential) patients between 50 and 80 years of age, with a Mini mental state examination (MMSE) score of 22 to 30, CSF biomarkers indicating AD pathology (p-tau >62 pg/ml, total CSF Aβ 1-42/1-40 ratio ≤0.055), a MRI scan in accordance with AD diagnosis not older than 3 months, at least 3 months stable medication prior to screening, were enrolled.
Recruitment	Patients randomized were prescreened at the Memory Clinic of the Charité Universtitätsmedizin Berlin (Germany), Department of Psychiatry and Neuroscience, which resulted in a low number of screening failures.
Ethics oversight	The study protocol and consent forms were approved by the local authorities (Landesamt für Gesundheit und Soziales, LaGeSo).

Note that full information on the approval of the study protocol must also be provided in the manuscript.

Field-specific reporting

Please select the one below that is the best fit for your research. If you are not sure, read the appropriate sections before making your selection.

[x] Life sciences [ ] Behavioural & social sciences [ ] Ecological, evolutionary & environmental sciences

For a reference copy of the document with all sections, see nature.com/documents/nr-reporting-summary-flat.pdf

Life sciences study design

All studies must disclose on these points even when the disclosure is negative.

Sample size	No sample size calculations were done because this trial was exploratory.
Data exclusions	No data were excluded
Replication	This trial was exploratory, so the findings were not replicated.
Randomization	Twenty patients, who met all inclusion criteria giving written informed consent were enrolled. Randomization was performed uniformly (block randomization, 1:1) and emergency letters were produced by the pharmacy of the Charité. One patient withdraw informed consent after screening before treatment was initiated. 9 patients were allocated to the verum group and received PRI-002, while consequently 10 patients received placebo treatment. There was no gender specific stratification implemented since no differences concerning efficacy and safety of the investigational product in this regard were expected. After data cleanup and database lock, the biostatistician was given access to the randomization code and the data were for the first time unblinded.
Blinding	All involved parties in this trial (patients, investigators, Sponsor personnel) were blinded to study treatment throughout the whole trial period. Capsules containing contraloid acetate and placebo were of identical appearance and were provided by the pharmacy of the Charité. The pharmacy of the Charité also performed the blinding procedures. The pharmacy of the Charité was responsible for the random list and emergency envelopes in order to ensure concealment of the blinding procedures.

Reporting for specific materials, systems and methods

We require information from authors about some types of materials, experimental systems and methods used in many studies. Here, indicate whether each material, system or method listed is relevant to your study. If you are not sure if a list item applies to your research, read the appropriate section before selecting a response.

Materials & experimental systems

n/a	Involved in the study	
☐	☒	Antibodies
☒	☐	Eukaryotic cell lines
☒	☐	Palaeontology and archaeology
☒	☐	Animals and other organisms
☐	☒	Clinical data
☒	☐	Dual use research of concern
☒	☐	Plants

Methods

n/a	Involved in the study	
☒	☐	ChIP-seq
☒	☐	Flow cytometry
☐	☒	MRI-based neuroimaging

Antibodies

Antibodies used	• Nab228: Supplier Sigma-Aldrich, Catalog number: A8354, Clone name: clone NAB 228, Lot: 019M4788V<br>• Tau5: Supplier Biolegend, Catalog number: 806403, Clone name: Tau5, Lot: B249294
Validation	• Nab228: https://www.uniprot.org/uniprotkb/P05067/entry<br>• Tau: https://www.uniprot.org/uniprotkb/?query=Tau 210-230

Clinical data

Policy information about clinical studies
All manuscripts should comply with the ICMJEguidelines for publication of clinical research and a completedCONSORT checklist must be included with all submissions.

Clinical trial registration	https://clinicaltrials.gov/study/NCT04711486; EudraCT 2020-003416-27
Study protocol	the study protocol was send to the Senior Editor Sophie Morgan of Nature Communications
Data collection	Data collection on each subject was recorded on a Case Report Form provided by the Principal Investigator. The original is kept at the trial centre. The study centre file all data electronically. To verify accuracy of the data, range, validity and consistency checks were performed. The first patient entered the study on 8 December 2020 (first patient in), and the last patient completed the follow-up visit at Day 56 on 13 January 2022 (last patient out). After data cleanup and database lock, the biostatistician was given access to the randomization code and the data were for the first time unblinded.
Outcomes	Primary objective of the study was the assessment of safety and tolerability of multiple oral doses of PRI-002 in patients with MCI or mild dementia due to AD. Primary endpoints included nature, frequency, severity, and timing of AEs and SAEs; changes in routine laboratory values, ECG, MRI, EEG, and vital signs. Secondary endpoints included the evaluation of pharmacokinetic characteristics of PRI-002 by determination of maximum plasma concentration (Cmax), time to reach maximum plasma concentration (Tmax), half-life (t1/2) calculated from PRI-002 plasma concentrations; and the determination of CSF concentrations of PRI-002.

Plants

Seed stocks	n/a
Novel plant genotypes	n/a
Authentication	n/a

Magnetic resonance imaging

Experimental design

Design type	structural MRI for safety reasons
Design specifications	2/3 per patient (before treatment, after treatment, at follow up)
Behavioral performance measures	n/a

Acquisition

Imaging type(s)	structural
Field strength	3 Tesla
Sequence & imaging parameters	sequences as recommended for safety
Area of acquisition	whole brain

Diffusion MRI ☐ Used ☒ Not used

Preprocessing

Preprocessing software	*Provide detail on software version and revision number and on specific parameters (model/functions, brain extraction, segmentation, smoothing kernel size, etc.).*
Normalization	*If data were normalized/standardized, describe the approach(es): specify linear or non-linear and define image types used for transformation OR indicate that data were not normalized and explain rationale for lack of normalization.*
Normalization template	*Describe the template used for normalization/transformation, specifying subject space or group standardized space (e.g. original Talairach, MNI305, ICBM152) OR indicate that the data were not normalized.*
Noise and artifact removal	*Describe your procedure(s) for artifact and structured noise removal, specifying motion parameters, tissue signals and physiological signals (heart rate, respiration).*
Volume censoring	*Define your software and/or method and criteria for volume censoring, and state the extent of such censoring.*

Statistical modeling & inference

Model type and settings	*Specify type (mass univariate, multivariate, RSA, predictive, etc.) and describe essential details of the model at the first and second levels (e.g. fixed, random or mixed effects; drift or auto-correlation).*
Effect(s) tested	*Define precise effect in terms of the task or stimulus conditions instead of psychological concepts and indicate whether ANOVA or factorial designs were used.*

Specify type of analysis: ☐ Whole brain ☐ ROI-based ☐ Both

Statistic type for inference (See Eklund et al. 2016)	*Specify voxel-wise or cluster-wise and report all relevant parameters for cluster-wise methods.*
Correction	*Describe the type of correction and how it is obtained for multiple comparisons (e.g. FWE, FDR, permutation or Monte Carlo).*

Models & analysis

n/a	Involved in the study
☒	☐ Functional and/or effective connectivity
☒	☐ Graph analysis
☒	☐ Multivariate modeling or predictive analysis
